# Stacking-fault strengthening of biomedical Co–Cr–Mo alloy via multipass thermomechanical processing

**DOI:** 10.1038/s41598-017-10305-1

**Published:** 2017-09-07

**Authors:** Kenta Yamanaka, Manami Mori, Shigeo Sato, Akihiko Chiba

**Affiliations:** 1 Institute for Materials Research, Tohoku University, 2-1-1 Katahira, Aoba-ku, Sendai, 980-8577 Japan; 2 Department of Materials and Environmental Engineering, National Institute of Technology, Sendai College, 48 Nodayama, Medeshima-Shiote, Natori, 981-1239 Japan; 3 Graduate School of Science and Engineering, Ibaraki University, 4-12-1 Nakanarusawa, Hitachi, 316-8511 Japan

## Abstract

The strengthening of metallic biomaterials, such as Co–Cr–Mo and titanium alloys, is of crucial importance to the improvement of the durability of orthopedic implants. In the present study, we successfully developed a face-centered cubic (fcc) Co–Cr–Mo alloy with an extremely high yield strength (1400 MPa) and good ductility (12%) by multipass hot-rolling, which is suitable for industrial production, and examined the relevant strengthening mechanisms. Using an X-ray diffraction line-profile analysis, we revealed that a substantial increase in the number of stacking faults (SFs) in the fcc γ-matrix occurred at a greater height reduction (*r*), while physical modeling demonstrated that the contribution of the accumulated SFs (i.e., the reduction in SF spacing) with an increase in *r* successfully explains the entire strengthening behavior of the hot-rolled alloy. The present study sheds light on the importance of the SF strengthening mechanism, and will help to guide the design and manufacturing strategy for the high-strength Co–Cr–Mo alloys used in highly durable medical devices.

## Introduction

Given their superior corrosion and wear resistance, Co–Cr–Mo alloys are widely used in orthopedic implants such as total hip replacements and spinal systems^1–4^. Recently, much attention has been paid to the strengthening of these alloys to improve the durability of such implants because existing metallic biomaterials often fail during clinical use^5, 6^. Furthermore, high-strength materials open avenues for novel designs of biomedical devices to improve patients’ quality of life (QOL); for instance, low-profile spinal-system devices with a reduced rod diameter may potentially be realized with high-strength Co–Cr–Mo alloys.

Thermomechanical processing to control the microstructure is one approach to the strengthening of Co–Cr–Mo alloys; therefore, many studies have addressed the hot deformation processing of these alloys^7–9^. Notably, we previously discovered dynamic recrystallization (DRX), which occurs in these alloys during hot deformation at temperatures of more than 1273 K^10–12^. It was revealed that DRX significantly reduces the grain size of the face-centered cubic (fcc) γ-matrix phase to a minimum grain size of 0.6 µm^10^, resulting in a very high tensile yield strength of 1400 MPa, which is much higher than that reported in the literature^11^.

The anomalously low stability of the γ-phase in Co–Cr–Mo alloys is the origin of the significant grain refinement due to DRX. The stacking fault energy (SFE) (*γ*_*SFE*_) is known to be closely related to the Gibbs energy difference between the fcc γ- and hexagonal close-packed (hcp) ε-phases^13^:

1 $${\gamma }_{SFE}=2\rho {\rm{\Delta }}{G}^{\gamma \to \varepsilon }+2{\sigma }^{\gamma /\varepsilon },$$

where Δ*G* ^*γ → ε*^ is the molar Gibbs energy change of the γ → ε phase transformation, *ρ* is the molar surface density along the {111} planes in the fcc structure, and *σ* ^*γ*/*ε*^ is the interfacial energy between the γ- and ε-phases. Because the equilibrium temperature between the γ- and ε-phases (*T*_0_ at Δ*G* ^*γ → ε*^ = 0) is relatively high (around 1173 K) compared to that of other metallic materials, Co–Cr–Mo alloys undergo a peculiar hot deformation under a considerably low SFE of approximately 50 mJ m^−2^, which is comparable to or slightly higher than the room-temperature SFE (e.g. 20.5–42 mJ m^−2^) of austenitic twinning-induced plasticity (TWIP) steels^14^, or typical low-SFE alloys (the SFE generally increases with temperature and reaches sufficiently high values at hot-deformation temperatures, e.g., > 200 mJ m^−2^ for TWIP steels^14^). This leads to strain accumulation due to the presence of highly planar dislocation structures even at elevated temperatures, which leads to DRX with significant grain refinement^12^. However, relatively large strains are necessary to obtain homogeneous UFG microstructures via DRX, which is the main obstacle preventing the application of a DRX-dominated strategy to industrial production using actual manufacturing processes (forging, rolling, drawing, etc.).

In this work, we focus on multipass thermomechanical processing, which involves the repeated introduction of relatively small amounts of deformation (strain) at elevated temperatures and which is suitable for industrial production. Owing to the anomalously low SFE of these alloys, we can expect a remarkable increase in the dislocation density as a result of the process. Furthermore, strengthening due to planar defects is anticipated with this process. It was recently reported that, in some Mg alloys with an hcp structure, the tensile strength can be improved by introducing stacking faults (SFs) with nanoscale spacing on their basal (0001) plane^15–18^. The following model was proposed to account for the contributions of the SFs (*σ*_*SF*_)^15, 16^:

2 $${\sigma }_{SF}\,=\,\frac{{k}_{SF}}{{L}_{SF}},$$

where *k*_*SF*_ is a constant and *L*_*SF*_ is the interspacing between SFs. Basal-SF-driven ultra-high strength was also reported in hcp titanium at the nanoscale^19^. For fcc metals and alloys, which include biomedical Co–Cr–Mo alloys, however, very few studies for quantitatively and directly evaluating the correlation between the SF formation and strength have been reported^20, 21^, although the strain-hardening behavior during tensile deformation has been well described in terms of SFE^22–26^. Moreover, unlike room-temperature deformation, a complicated microstructural evolution, which involves not only the introduction of lattice defects (dislocations/SFs) but also thermally activated processes such as DRX with grain refining, would occur at high temperatures. Therefore, a comprehensive study examining the microstructure development and its effect on the overall strengthening would be highly desirable to optimize its processing and properties and to obtain a deeper understanding of its microstructure–property relationships.

In this study, we performed multipass hot-rolling of a Co–Cr–Mo alloy and examined the tensile properties and hot-rolled microstructures in terms of the accumulated strain. It is difficult to quantitatively characterize the behaviors of dislocations and SFs in highly deformed microstructures by transmission electron microscopy (TEM). X-ray diffraction (XRD) line-profile analyses were therefore employed to study the evolution of the dislocation density and the number of SFs present during the process. On the basis of the generated data, we analyzed the contributions of the relevant strengthening mechanisms to illustrate how SFs add to the overall strength (in combination with other strengthening mechanisms) so that they may be used in an even wider application range in fcc alloys.

## Results

### Tensile properties of prepared hot-rolled specimens

**Figure 1:**
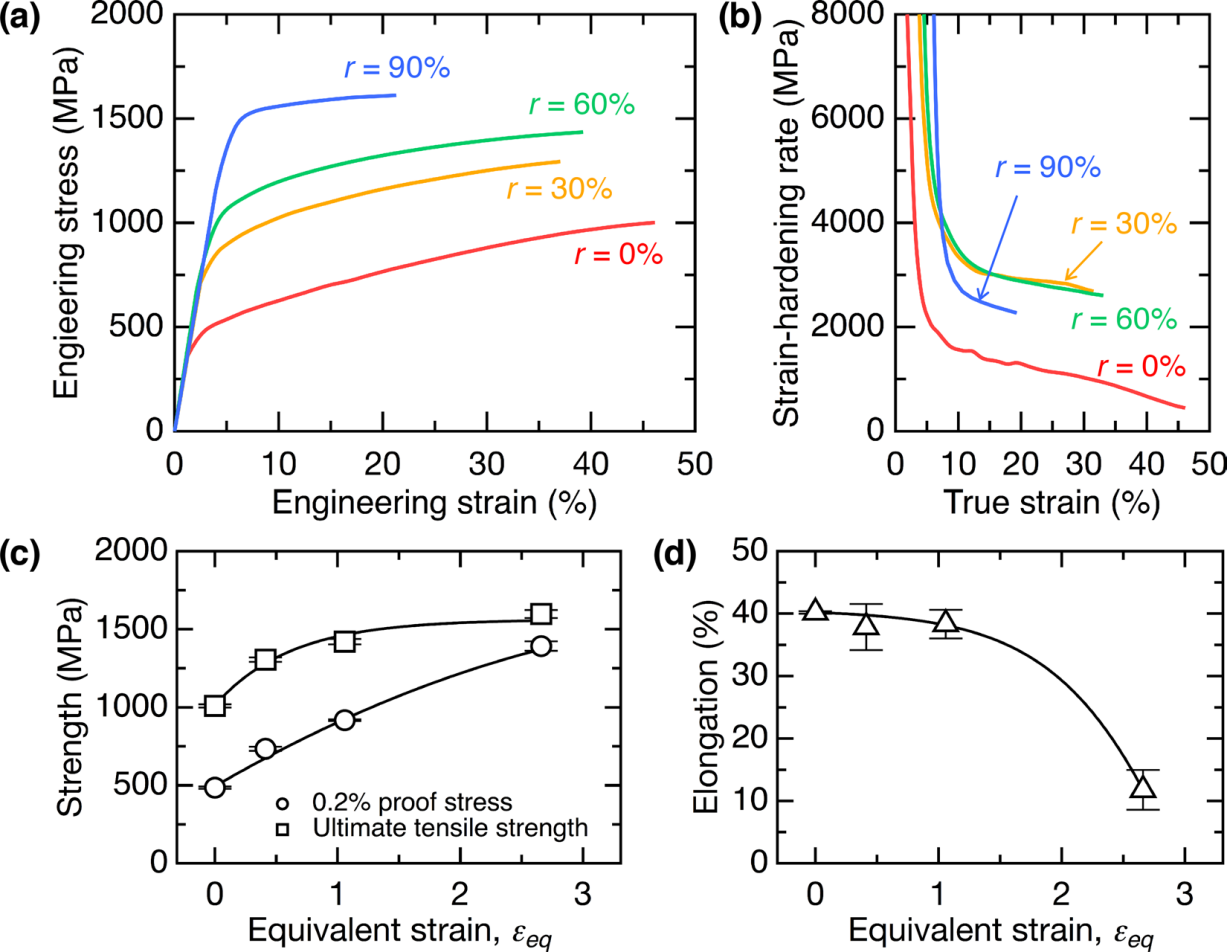
Mechanical properties of a Co–Cr–Mo alloy after multipass hot-rolling. (**a**) Engineering stress–strain curves obtained by tensile testing along the rolling direction (RD) and (**b**) corresponding strain-hardening curves with respect to the true strain of the hot-rolled specimens. (**c**) Strength and (**d**) elongation-to-failure results plotted as functions of equivalent strain (*ε*_*eq*_) imposed during the multipass hot-rolling process.

Figure 1(a) shows the tensile stress–strain curves of the initial solution-treated specimen and those specimens that were hot-rolled to a cumulative height reduction (*r*) of up to 90%. They were obtained by tensile loading along the rolling direction (RD). The specimens exhibited apparent strain hardening and subsequent fractures without any clear macroscopic necking, which is typical of Co–Cr–Mo alloys. Figure 1(b) shows the corresponding strain-hardening behavior with respect to the true strain. Interestingly, the *r* = 30% and *r* = 60% specimens show almost identical strain-hardening rates, these being significantly higher than that of the initial specimen over the entire loading range. The strain-hardening rate of the *r* = 90% specimen, despite the remarkable increase in strength, was still higher than that of the initial specimen, although it decreased slightly from those of the hot-rolled specimens processed with the lower value of *r*. Figure 1(c,d) plots the tensile strength and elongation-to-failure of the prepared specimens as functions of equivalent strain (*ε*_*eq*_) imposed during the multipass hot-rolling process. The initial specimen had a 0.2% proof stress of 485 MPa and a large elongation-to-failure of approximately 40%. The 0.2% proof stress increased monotonically with *ε*_*eq*_, while the ultimate tensile strength became saturated at higher *ε*_*eq*_ values. The elongation-to-failure (uniform elongation) was found to decrease with an increase in *ε*_*eq*_, although those specimens that were hot-rolled up to *ε*_*eq*_ = 1.06 (*r* = 60%) exhibited an almost identical ductility. However, a remarkably high 0.2% proof stress of around 1400 MPa and a good elongation-to-failure of 12% were obtained at *ε*_*eq*_ = 2.66 (*r* = 90%). These results demonstrate that the present industrially feasible multipass processing method can realize a superior strength-ductility balance that is comparable to that of DRX-mediated UFG materials^11^.

### Microstructural development during processing

**Figure 2:**
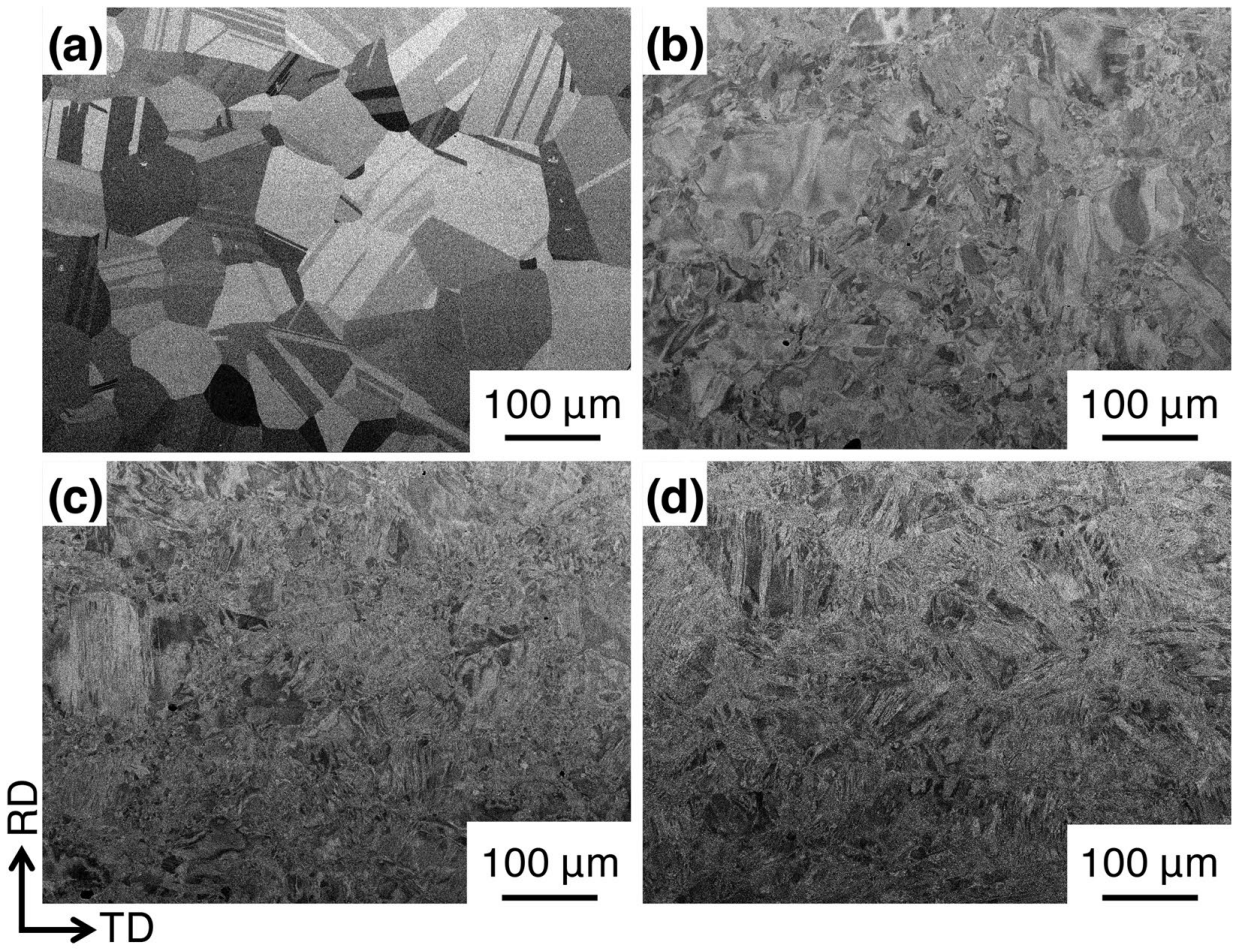
Scanning electron microscopy-backscattered electron (SEM-BSE) images of multipass hot-rolled specimens. (**a**) Initial specimen and specimens hot-rolled to an accumulative height reduction (*r*) of (**b**) 30%, (**c**) 60%, and (**d**) 90%.

The scanning electron microscopy-backscattered electron (SEM-BSE) image of the initial microstructure prior to hot-rolling, shown in Fig. 2(a), reveals a uniform equiaxed grain structure with annealing-twin boundaries (ATBs). In contrast, the morphology of the grains became unclear with an increase in *r* and was difficult to identify beyond *r* = 90% (Fig. 2(b–d)). We did not find precipitates (e.g., σ-phase and M_23_C_6_ carbide) in any of the specimens.

**Figure 3:**
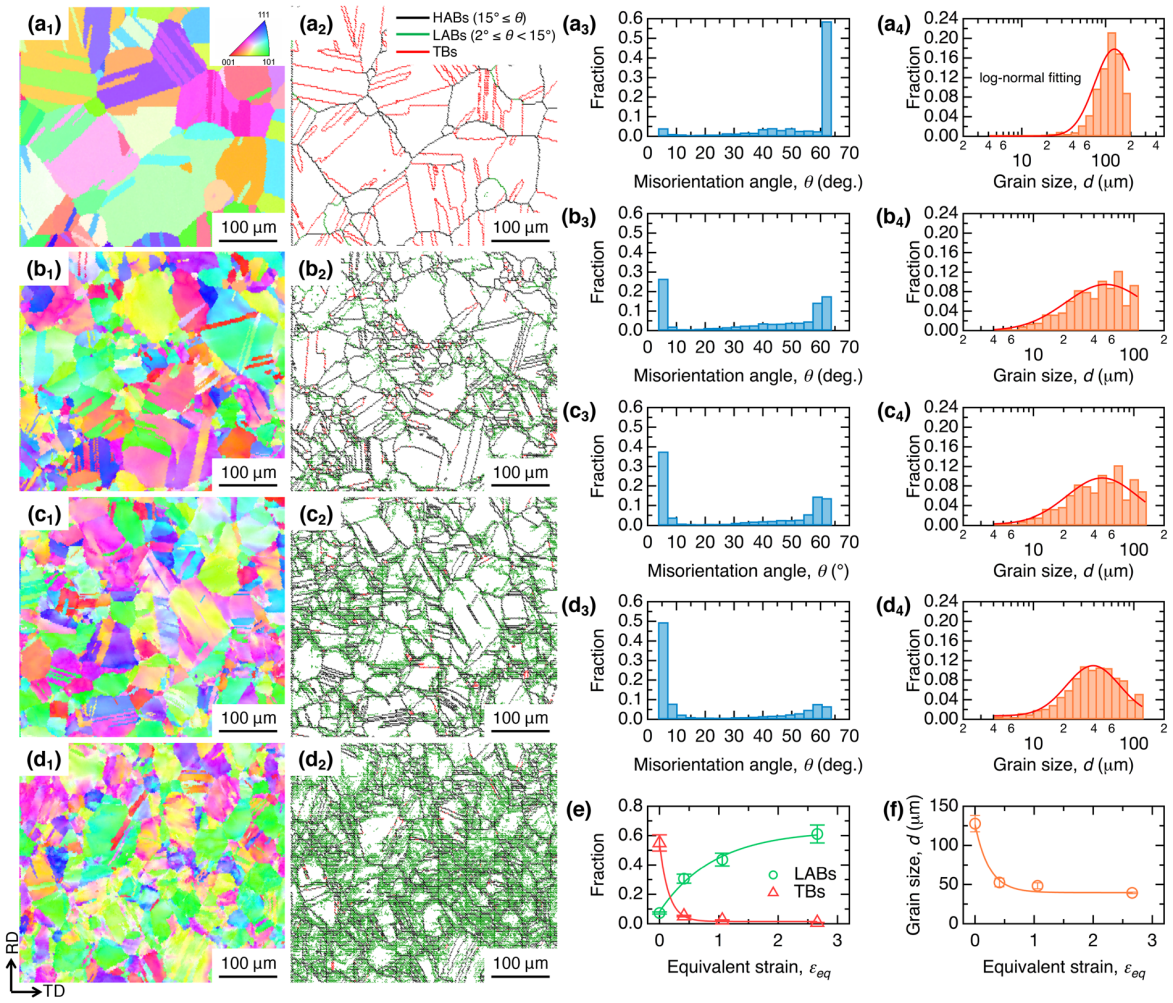
Electron backscatter diffraction (EBSD) maps for γ-phase of initial and hot-rolled specimens. (**a**_**1**_–**d**_**1**_) Inverse pole figure (IPF) maps, (**a**_**2**_–**d**_**2**_) boundary maps, and the corresponding (**a**_**3**_–**d**_**3**_) misorientation and (**a**_**4**_–**d**_**4**_) grain size distributions for the (**a**_**1**_–**a**_**4**_) initial specimen and specimens hot-rolled to an accumulative height reduction (*r*) of (**b**_**1**_–**b**_**4**_) 30%, (**c**_**1**_–**c**_**4**_) 60%, and (**d**_**1**_–**d**_**4**_) 90%. The black, green, and red lines in the boundary maps indicate high-angle boundaries (HABs) with misorientation angles larger than 15°, low-angle boundaries (LABs) with misorientation angles of 2–15°, and Σ3 twin boundaries (TBs), respectively. Evolution of (**e**) LABs and TBs and (**f**) average grain size (***d***) as a function of the equivalent strain (*ε*_*eq*_) are shown.

Figure 3 shows the results of the electron backscatter diffraction (EBSD) analysis of the specimens before and after multipass hot-rolling. Because 0.13 wt% of nitrogen incorporated into the alloy as a γ-phase stabilizing element sufficiently suppressed the athermal γ → ε martensitic transformation upon cooling after hot rolling^27^, only the fcc γ-phase was indexed in all the specimens, irrespective of the imposed *r*. The inverse pole figure (IPF) map of the initial microstructure exhibits uniform crystallographic orientations in each grain (Fig. 3(a_1_)). In contrast, in-grain misorientation was observed in the IPF maps of the hot-rolled specimens, and the degree of misorientation increased with *r* (Fig. 3(b_1_–d_1_)). The boundary maps and corresponding misorientation distributions are shown in Fig. 3(a_2_–d_2_) and Fig. 3(a_3_–d_3_), respectively. The initial specimen (Fig. 3(a_2_)) had many ATBs. The peak intensity at a misorientation of 60° in the misorientation distribution in Fig. 3(a_3_) indicates that approximately 55% of the boundaries in the initial sample corresponded to twin boundaries (TBs). The fraction of TBs significantly decreased upon hot-rolling; in contrast, the quantity of low-angle boundaries (LABs) increased upon hot-rolling, as shown in Fig. 3(e). A TB fraction of only 5% or less and a significant fraction of LABs (i.e., > 30%) were observed at *ε*_*eq*_ = 2.66 (*r* = 90%). Figure 3(a_4_–d_4_) shows the grain-size distributions of the specimens at each *r*. Figure 3(f) shows the average grain size (*d*) for each specimen, determined by log-normal fitting of the EBSD data without considering TBs. Grain refinement with increasing *ε*_*eq*_ was identified: the initial grain size of 128 μm was decreased to 53 μm at *ε*_*eq*_ = 0.41 (*r* = 30%) and then gradually decreased to 39 μm at *ε*_*eq*_ = 2.66 (*r* = 90%). A similar tendency was observed when TBs were taken into account in the grain-size calculations. These results indicate that grain refinement due to DRX and the development of deformation structures occurred simultaneously during the multipass hot-rolling.

**Figure 4:**
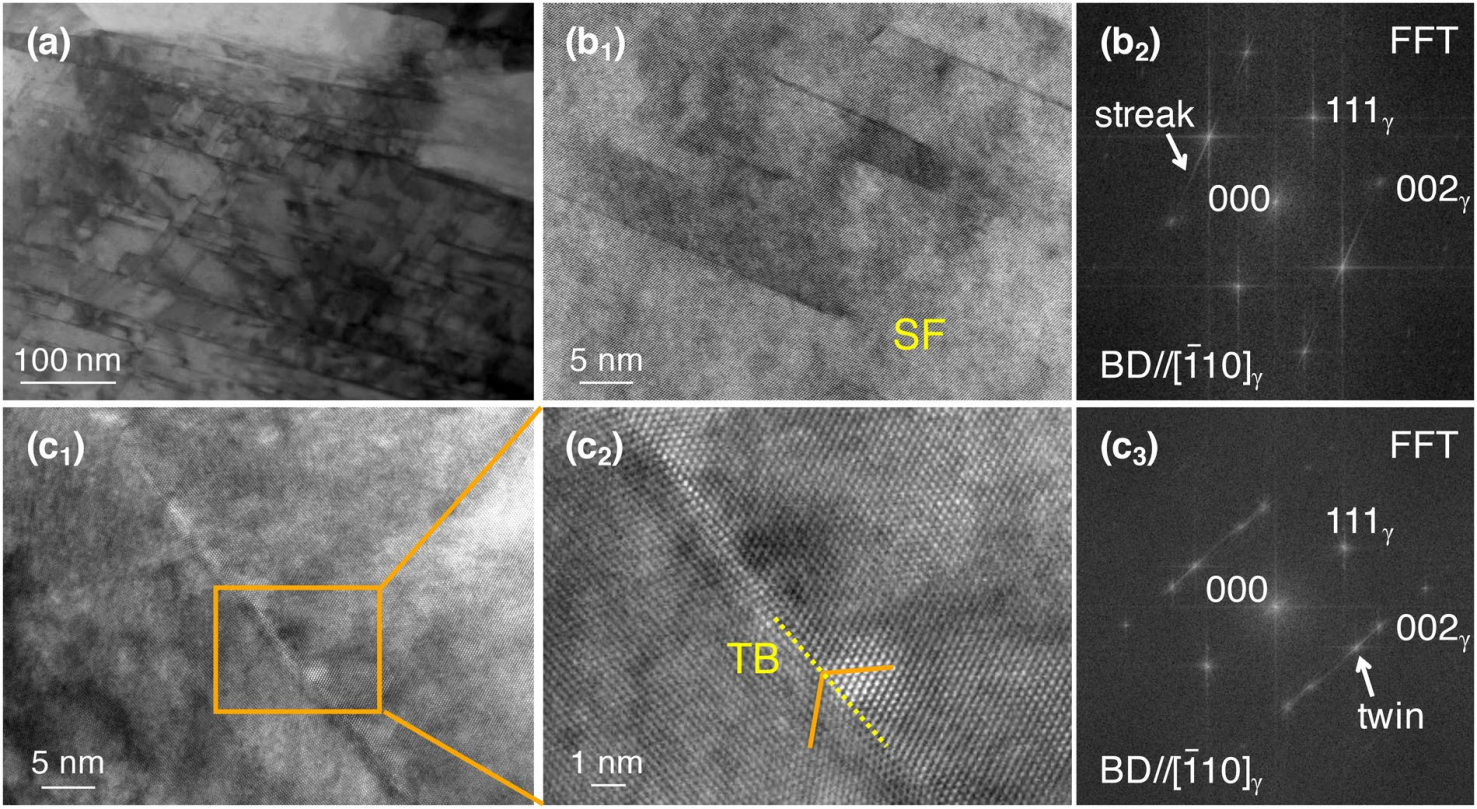
(High-resolution) transmission electron microscopy ((HR)TEM) of specimens hot-rolled to an accumulative height reduction of *r* = 90%. (**a**) TEM bright-field image and (**b**_**1**_,**c**_**1**_) HRTEM images. (**c**_**2**_) Magnified image of (**c**_**1**_). The fast Fourier transform (FFT) patterns of (**b**_**1**_) and (**c**_**2**_) are shown in (**b**_**2**_) and (**c**_**3**_), respectively.

Figure 4(a) shows a TEM bright-field image of the *r* = 90% specimen. A large number of planar defects, which intersect with each other, were found inside the γ-grains. High-resolution TEM (HRTEM) images and the corresponding fast Fourier transform (FFT) images (with the zone axis parallel to the [$\bar{1}\text{10}$]_γ_ direction) of the same specimen are shown in Fig. 4(b,c). The planar defects observed in Fig. 4(b_1_) produced streaks formed along the <111> _γ_ direction in the FFT image (Fig. 4(b_2_)), indicating the formation of SFs in the γ-matrix during the process. The interspacing of SFs was approximately 10 nm. In contrast, the interface shown in Fig. 4(c_1_,c_2_) showed extra diffraction spots, which arise from TB, in the corresponding FFT image (Fig. 4(c_3_). This indicates that deformation twinning (DT) occurred during the multipass hot-rolling, although EBSD could not index these nano-spaced twins owing to the resolution limit (Fig. 2(d_2_)). This is reasonable because the hot deformation of the present alloy occurs at an SFE comparable to those of TWIP steels at room temperature, as described in the Introduction.

### X-ray diffraction (XRD) line-profile analysis

**Figure 5:**
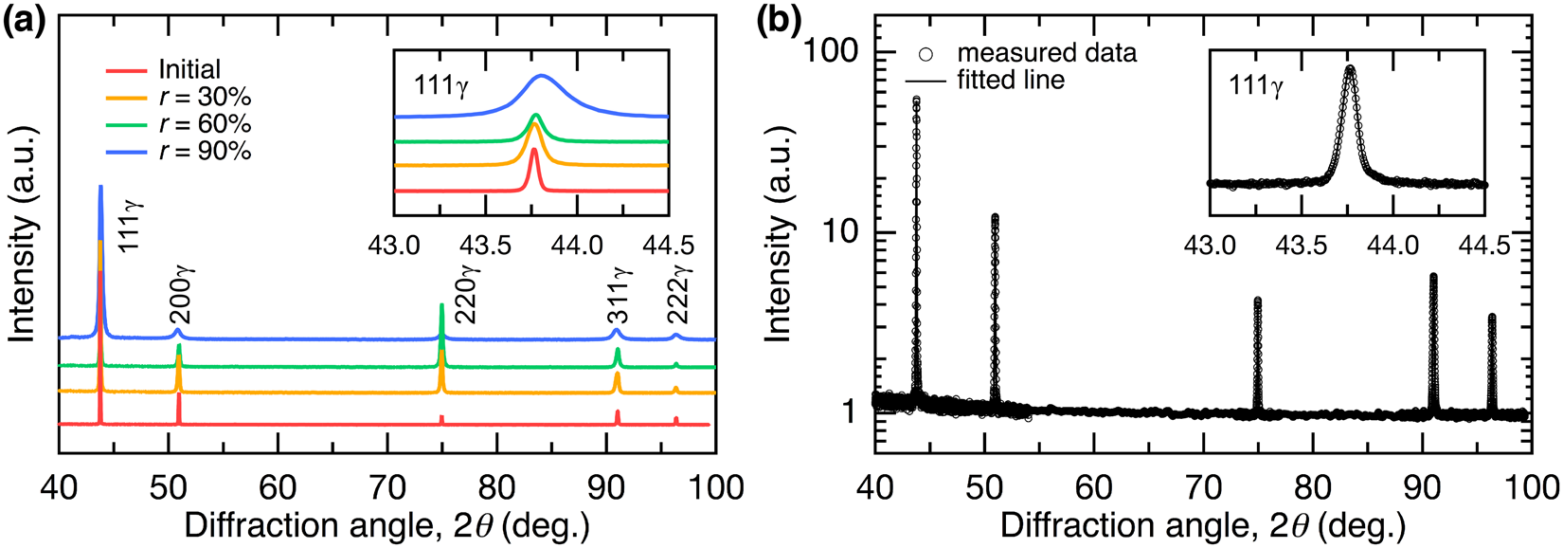
X-ray diffraction (XRD) analysis and extended convolutional multiple whole profile (eCMWP) fitting. (**a**) XRD patterns for each specimen and (**b**) an example of the eCMWP fitting result for the initial specimen. The open circles and the solid line represent the measured data and the theoretical curves obtained by eCMWP fitting, respectively. The intensity is plotted in logarithmic scale. The inset shows part of the diffractogram at higher magnification.

Figure 5(a) shows the XRD patterns obtained from the specimens before and after hot-rolling. Similar to the EBSD results, all of the specimens exhibited only fcc γ-peaks. The peak broadening induced by hot-rolling was clearly observed (see inset). XRD line-profile analysis based on the extended convolutional whole profile (eCMWP) fitting^28, 29^ was performed on the measured profiles. The eCMWP method was developed based on the conventional CMWP fitting method^30, 31^, and can be used to examine the evolution of the “SF probability” (SFP), in addition to dislocation structures. Figure 5(b) shows a typical example of the eCMWP fitting results for the initial specimen. The eCMWP fitting was conducted for the 111, 200, 220, 311, and 222 peaks of the γ-phase of each specimen. Although the initial specimen without deformation had very narrow diffraction peaks, which often made the fitting procedure difficult, the measured line profile (open symbols) fitted the eCMWP procedure (solid line) well.

**Figure 6:**
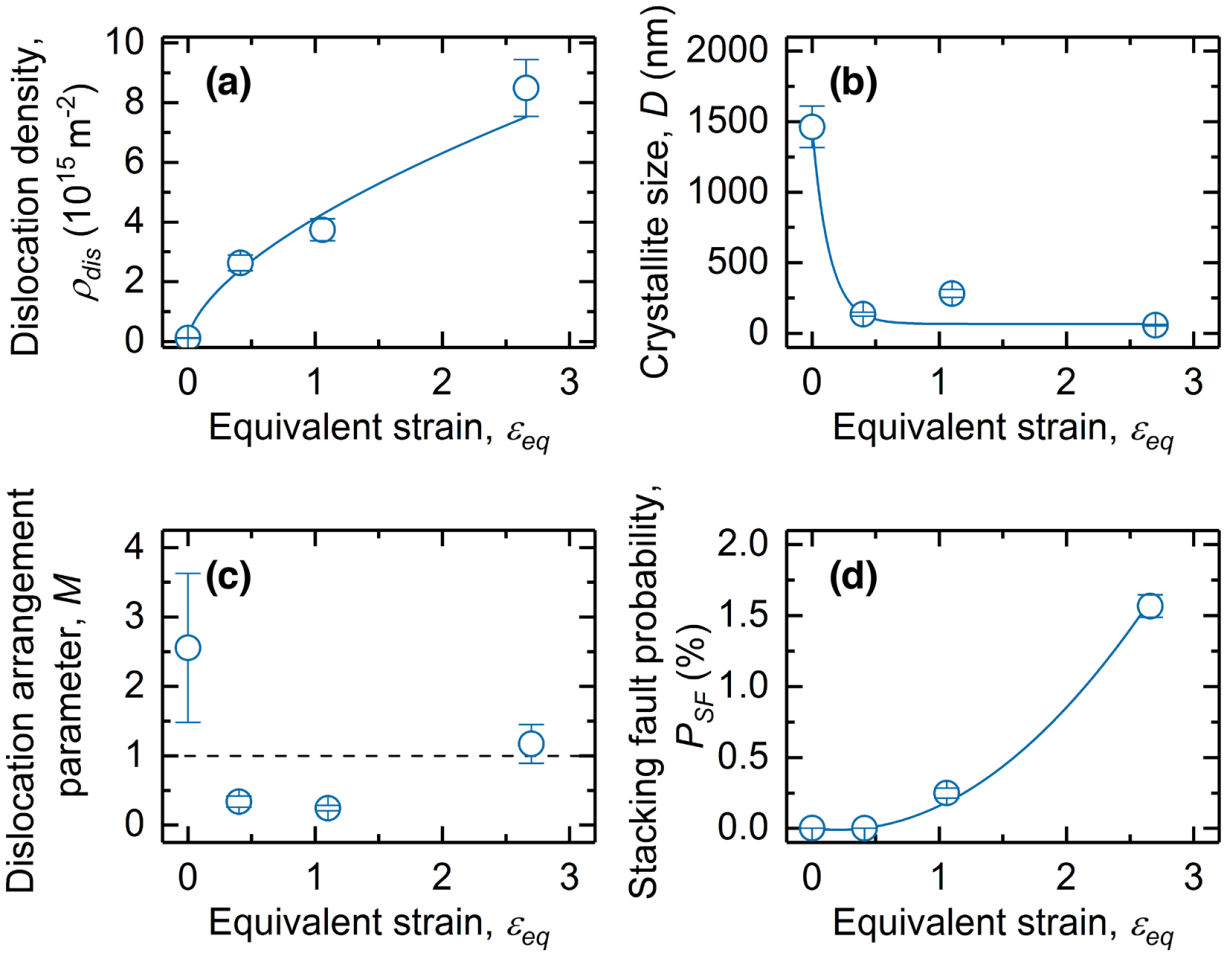
Results of extended convolutional multiple whole profile (eCMWP) analysis of hot-rolled specimens. (**a**) Dislocation density (*ρ*_*dis*_), (**b**) crystallite size (*D*), (**c**) dislocation arrangement parameter (*M*), and (**d**) stacking fault probability (SFP) (*P*_*SF*_) obtained by X-ray diffraction (XRD) line-profile analyses, based on the eCMWP fitting of the hot-rolled specimens as a function of the equivalent strain (*ε*_*eq*_).

Figure 6 shows the results of the eCMWP fitting analysis of the prepared specimens, plotted as a function of *ε*_*eq*_. As shown in Fig. 6(a), the dislocation density (*ρ*_*dis*_) for Shockley partial dislocations was 1.2 × 10^14^ m^–2^ for the initial solution-treated specimen. The *ρ*_*dis*_ value first strongly increased with *ε*_*eq*_ and then became saturated at higher values of *r*. However, a very high dislocation density of 8.5 × 10^15^ m^−2^, which is almost two orders of magnitude higher than that of the initial specimen, was obtained for *ε*_*eq*_ = 2.66 (*r* = 90%).

Figure 6(b) shows the evolution of the mean crystallite size (*D*), which defines the size of domains that coherently diffract incident X-rays^30^ during the hot-rolling process. The crystallite size for the initial specimen (*D* = 1462 nm) significantly decreased at the beginning of the hot-rolling (i.e., *ε*_*eq*_ = 0.41 (*r* = 30%)), which correlates to the increase in the dislocation density. The *D* value then decreased slightly to approximately 50 nm via further deformation.

Figure 6(c) shows the dislocation arrangement parameter *M* as a function of *ε*_*eq*_. *M* values that are smaller or larger than unity indicate a strong or weak interaction between dislocations, respectively^32^. The initial specimens had an *M* value well above 1, while *M* decreased to less than 1 at *ε*_*eq*_ = 0.41 (*r* = 30%). A similarly low *M* value was obtained at *ε*_*eq*_ = 1.06 (*r* = 60%). The decrease in *M* indicates that the dislocations introduced during hot-rolling were rearranged into low-energy configurations. This may be explained by the development of LABs, as captured with EBSD analyses (Fig. 3). Further deformation (i.e., *r* = 90%) slightly increased *M*, indicating that newly formed excess lattice defects were introduced in the interiors surrounded by LABs in the later stage of the multipass hot-rolling process.

Finally, Fig. 6(d) shows the variation in the SFP (*P*_*SF*_) as a function of *ε*_*eq*_. The eCMWP fitting procedure clearly captured the evolution of the SFs during the multipass hot-rolling process. *P*_*SF*_ was almost zero up to *ε*_*eq*_ = 0.41 (*r* = 30%), at which point it increased sharply with further increase in *ε*_*eq*_. *P*_*SF*_ was substantially higher at *ε*_*eq*_ = 2.66 (*r* = 90%), reaching approximately 1.6%, which indicates its dramatic influence on the mechanical behavior of the alloy. This result quantitatively demonstrates our hypothesis that a significant accumulation of SFs occurs even at high temperatures (e.g., 1473 K) if the alloy has a sufficiently low SFE.

## Discussion

**Figure 7:**
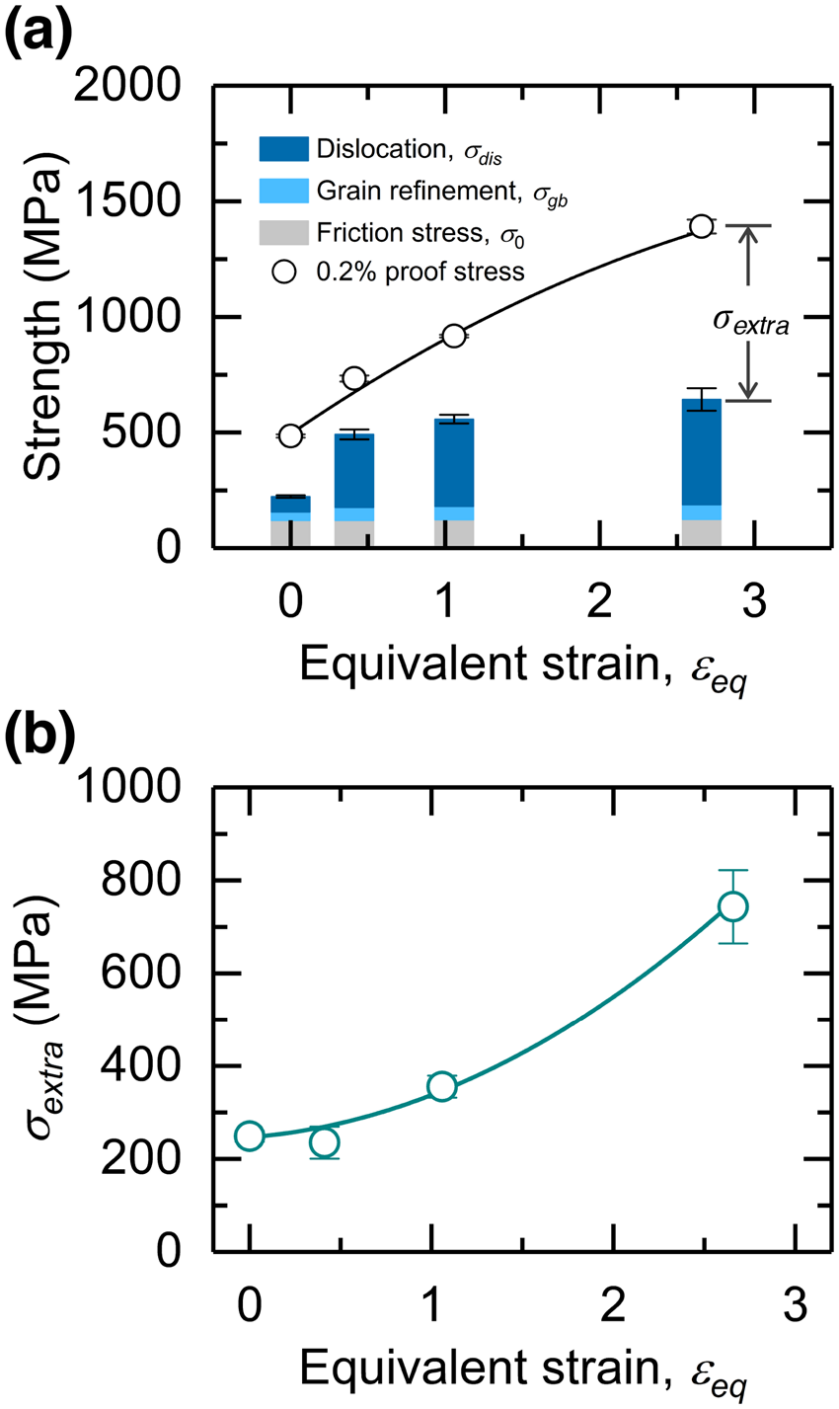
Relationship between accumulated height reduction (*r*), calculated and experimentally measured yield and proof stress, and extra strengthening. (**a**) Comparison between the calculated yield stress (*σ*_*y,calc*_) and experimentally obtained 0.2% proof stress (*σ*_0.2_) as a function of *r*, and (**b**) relationship between the extra strengthening (*σ*_*extra*_, as defined in Eq. (6)) and *r*.

In general, there are four strengthening mechanisms at work in metallic materials^33^: solid-solution hardening, precipitation hardening, dislocation hardening, and grain refinement. The contributions of grain refinement (*σ*_*gb*_) and dislocation density (*σ*_*dis*_) are expressed by the Hall–Petch and Bailey–Hirsch (or Taylor) relationships, respectively:

3 $${\sigma }_{gb}=k{d}^{-1/2},$$

4 $${\sigma }_{dis}={M}_{Taylor}\alpha Gb{\rho }_{dis}^{1/2},$$

where *k* is the Hall–Petch coefficient, *d* is the grain size, *M*_*Taylor*_ is the Taylor factor, *α* is a constant, *G* is the shear modulus, and *b* is the value of the Burgers vector. In the present study, the hot-rolled specimens were prepared from the same alloy ingot, and we did not identify the secondary phase precipitation in any of the specimens (Fig. 2). Therefore, it is likely that the solid-solution hardening and precipitation hardening do not create a significant difference in the strengths of the prepared specimens. Thus, we defined the “calculated yield stress” (*σ*_*y,calc*_) as follows:

5 $${\sigma }_{y,calc}={\sigma }_{0}+{\sigma }_{gb}+{\sigma }_{dis},$$

where *σ*_0_ is the friction stress. By summing the obtained results, *σ*_*y,calc*_ for the prepared hot-rolled specimens was calculated and is plotted as a function of *ε*_*eq*_ in Fig. 7(a). Table 1 presents the parameters used in the calculations^34–37^. The value of the Burgers vector (*b*_*p*_) for Shockley partial dislocation was employed to determine the *σ*_*dis*_ values. The *σ*_0_ values (around 120 MPa) can be calculated using the Taylor factor (*M*_*Taylor*_) and the critical resolved shear stress (*τ*_*CRSS*_ = 54 MPa^36^), where *σ*_0_ = *M*_*Taylor*_ • *τ*_*CRSS*_. The *M*_*Taylor*_ value was obtained for each specimen based on the results of the EBSD measurements; thus, the effect of the hot-rolling texture on the strength of the specimens could be incorporated into the analyses, although negligible anisotropy was found in the prepared specimens. These results indicate that the contribution of the grain-boundary (GB) strengthening increased from 35 MPa for the initial specimen to 64 MPa for *ε*_*eq*_ = 2.66 (*r* = 90%). The increased dislocation density plays a more significant role in the overall strength, and its contribution increases remarkably with *ε*_*eq*_; *σ*_*dis*_ was calculated to be 455 MPa at *ε*_*eq*_ = 2.66 (*r* = 90%). However, the obtained *σ*_*y,calc*_ values were still much lower than the experimentally measured 0.2% proof stresses (*σ*_0.2_). Therefore, we defined this discrepancy as the “extra strengthening” (*σ*_*extra*_).

6 $${\sigma }_{extra}={\sigma }_{0.2}-{\sigma }_{y,calc}={\sigma }_{0.2}-({\sigma }_{0}+{\sigma }_{gb}+{\sigma }_{dis}).$$

**Table 1:** Parameters for obtaining calculated yield stresses (*σ*_*y,calc*_) of hot-rolled specimens.

Parameter	Value
Shear modulus, *G* (GPa)	78.4^34^
Burgers vector of Shockley partial dislocations, *b*_*p*_ (nm)	0.1463^34^
*α*	0.24^35^
Critical resolved shear stress, *τ*_*CRSS*_ (MPa)	54^36^
Hall–Petch coefficient, *k* (MPa m^1/2^)	0.4^37^
Taylor factor (determined by EBSD), *M*_*Taylor*_	2.24 (*r* = 0%)
	2.24 (*r* = 30%)
	2.29 (*r* = 60%)
	2.31 (*r* = 90%)

Interestingly, the *σ*_*extra*_ values show an accelerated increase with an increase in *ε*_*eq*_ (Fig. 7(b)). *σ*_*extra*_ was approximately 743 MPa for the *r* = 90% (*ε*_*eq*_ = 2.66) specimen, which is quite significant relative to the corresponding *σ*_0.2_. Therefore, we can conclude that the strengthening obtained by the multipass hot-rolling process cannot be explained only in terms of the grain refinement and accumulated dislocations.

In contrast, in the present study, we discovered that the SFP (*P*_*SF*_) dramatically increased, particularly at greater values of *r* (Fig. 6(d)). This indicates that plastic deformation in multipass hot rolling was predominantly accommodated by Shockley partial dislocations to generate SFs. A transition from perfect to partial dislocations was previously reported when the grain sizes and/or plastic deformation lie on nanoscale regimes^38–40^. In the present study, however, we used Co–Cr–Mo alloys with grain sizes of at least several tens of micrometers. Therefore, we were not able to clarify such a transition. Here, the relationship between the “extra strengthening” and the accumulated SFs is discussed. The *P*_*SF*_ value, which was obtained by the eCMWP fitting procedure, can be defined as the frequency of SFs formed along the {111} plane of the γ-phase. Therefore, the “average value” of *L*_*SF*_ can be determined using *P*_*SF*_ (in percentage):

7 $${L}_{SF}={d}_{111}/(\frac{{P}_{SF}}{100}),$$

where *d*_111_ is the interspacing between the {111}_γ_ planes.

**Figure 8:**
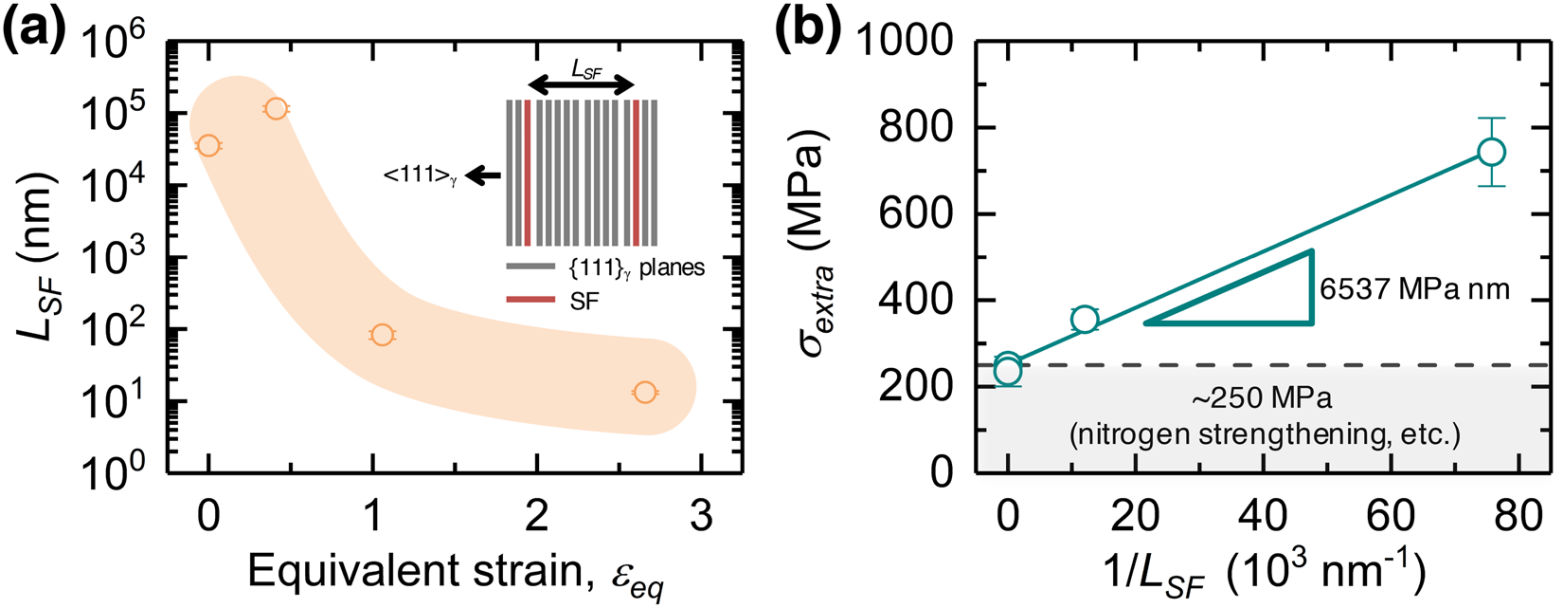
Effect of stacking fault (SF) spacing on mechanical properties of multipass hot-rolled Co–Cr–Mo alloy. (**a**) Evolution of interspacing between SFs (*L*_*SF*_) as a function of the equivalent strain (*ε*_*eq*_). (**b**) Extra strengthening (*σ*_*extra*_), as defined in Eq. (6), as a function of *L*_*SF*_.

Figure 8(a) summarizes the *L*_*SF*_ values determined from *P*_*SF*_ as a function of *ε*_*eq*_. *L*_*SF*_ was more than several tens of micrometers in the initial and *r* = 30% (*ε*_*eq*_ = 0.41) specimens, indicating that very few SFs existed at the early stage of the multipass processing. However, the *L*_*SF*_ value decreased dramatically with an increase in *ε*_*eq*_, and a very small *L*_*SF*_ of approximately 10 nm was obtained after the 90% hot-rolling. The *L*_*SF*_ value obtained here is in good agreement with that obtained from TEM observations (Fig. 4(b)), indicating that the present eCMWP method can provide reasonable quantitative evaluations of the *L*_*SF*_ values.

Figure 8(b) plots the extra strengthening (*σ*_*extra*_) as a function of 1/*L*_*SF*_ in accordance with Eq. (2). A linear relationship was obtained, indicating that the extra strengthening defined above can be well described in terms of the model proposed by Jian *et al*.^15, 16^. Therefore, we can conclude that the SFs introduced during multipass hot-rolling contribute to the evolution of the extra strengthening. The intercept at 1/*L*_*SF*_ = 0 is not zero (around 250 MPa), indicating that the extra strengthening does not originate solely from the accumulated SFs and that there are contributions from other than the above-mentioned mechanisms. Nitrogen doping may be a dominant contributing factor for this because it can increase the yield stress by 100–200 MPa^27^. Thus, the contribution of the SF strengthening is as high as 500 MPa at *ε*_*eq*_ = 2.66 (*r* = 90%), which is quite significant relative to the overall strengthening due to multipass hot-rolling.

The physical model described by Eq. (2) was originally proposed for hcp Mg alloys with parallelly spaced SFs on their basal planes^15, 16^. To the best of our knowledge, the present study is the first to demonstrate that the model is applicable to fcc metals and alloys. It is interesting to note that the slope of the linear relationship (*k*_*SF*_) in Fig. 8(b) is 6537 ± 342 MPa nm, which is of the same order but much larger than that obtained for a Mg alloy (*k*_*SF*_ = 3780 MPa nm). In the model, *k*_*SF*_ can be expressed as a function of the extra energy consumed by cutting through SFs when dislocations are impinged at the SFs^16^. Therefore, the large *k*_*SF*_ value of the present alloy indicates that, compared with Mg alloys, SFs in fcc metals and alloys can act as a strong obstacle for dislocation gliding. Because SFs in the fcc crystals can form on all four {111} planes (i.e., (111), $(\overline{1}11)$, $(1\overline{1}1)$, and $(11\overline{1})$), intersecting SFs occur during plastic deformation, as shown in Fig. 4(a). It was suggested from geometric arguments that the intersection of two SFs acts as a “dislocation dipole”^41^, providing a strong obstacle to dislocation, whereas SFs themselves are not strong barriers^42^. Therefore, the SF intersections resulting from multiple activations of SFs on different {111} planes during the process would play a considerable role in this strengthening mechanism compared with Mg alloys, in which SFs form almost parallel to each other on the basal (0001) plane. Further investigations are required to clarify these interactions.

**Figure 9:**
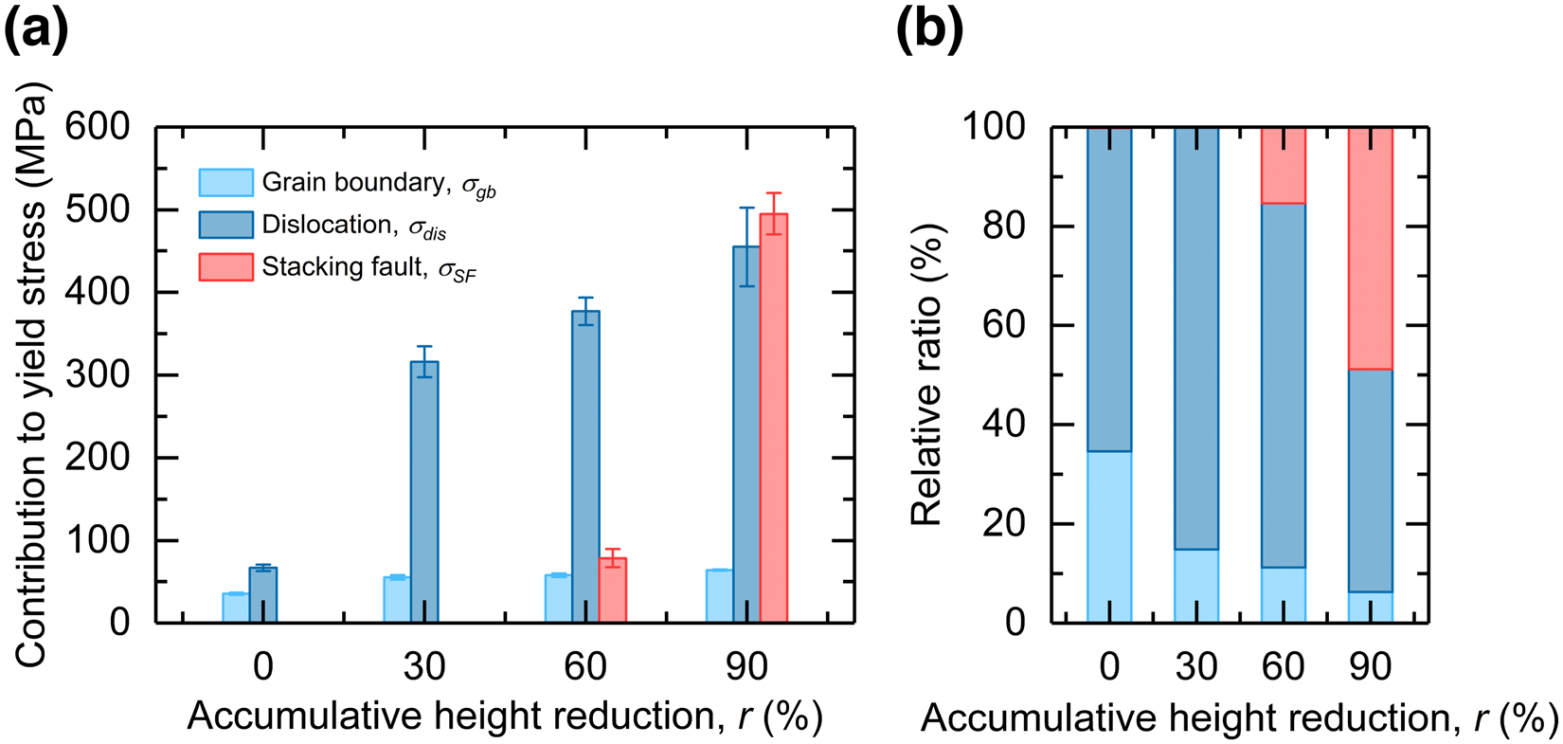
Resolved effects of microstructure components on mechanical properties of multipass hot-rolled Co–Cr–Mo alloy. (**a**) Contributions of grain boundaries (*σ*_*gb*_), dislocations (*σ*_*dis*_), and stacking faults (SFs) (*σ*_*SF*_) to the yield stress, and (**b**) their relative ratio to the overall strengthening effect in the prepared hot-rolled specimens.

Using the *k*_*SF*_ value determined here, we ascertained the contributions of grain refinement (*σ*_*gb*_), dislocations (*σ*_*dis*_), and SFs (*σ*_*SF*_), all of which were achieved by multipass hot-rolling, in the prepared specimens at different values of *r* (Fig. 9(a)). The *σ*_*gb*_ values were as high as 50 MPa, irrespective of *r*. The increased dislocation density contributed to the yield stress significantly; the *σ*_*dis*_ values increased steeply between the initial condition and *r* = 30%, and dislocations accounted for more than 80% of the strengthening effect caused by multipass hot-deformation in the specimens deformed up to *r* = 60% (Fig. 9(b)). In contrast, the SF strengthening was not evident in the initial and *r* = 30% specimens, but became significant at greater *r*. Surprisingly, the contribution of the SFs at *r* = 90% (around 500 MPa) is comparable or even superior to that from the dislocations. Thus, the increase in the SF width, which is preferred under a large external stress^43^, suitably explains the *r*-dependence of *σ*_*SF*_. These results shed light on the importance of the SF strengthening, which has not yet been directly considered, and will help in guiding the design and manufacturing strategy for high-strength fcc alloys using lattice defects. Furthermore, the eCMWP-based XRD line-profile analysis employed in this study is a powerful technique capable of statistically determining SF densities and modeling the contribution to the strength.

It is also noteworthy that the formation of ATBs and DTs was observed in the initial solution-treated (Fig. 3(a_2_)) and hot-rolled specimens (Fig. 4(c_1_–c_3_)), respectively. Recent studies have reported that TBs play an important role in the strengthening of metals^33, 44–47^. It was shown that the twin thickness (i.e., the spacing between two adjoining TBs) affects the yield strength in the same way as the grain boundaries, i.e., they conform to a Hall–Petch relationship^33, 46^. Although the Hall–Petch coefficient for TBs, which is similar to^33^ or often one order of magnitude smaller than that for grain boundaries^48, 49^, in these alloys is unknown at present, the contribution of ATBs was found to be minimal (no more than around 10 MPa) when ATBs were taken into account in the grain-size calculation using the EBSD results. Furthermore, careful TEM observations identified that the fraction of nanoscale DTs was not high in the present hot-rolled specimens; the fact that the extra strengthening defined above fully complied with Eq. (2), as shown in Fig. 8(b), implies that the formation of SFs was dominant over DT and that the effect of DTs on the strength is almost negligible in the present hot-rolled specimens. It is well known that there are critical stresses leading to the initiation of DT^43, 50, 51^ as well as DRX^52–54^. The critical stress for DT is regarded as being higher than that for DRX, as grain refinement due to DRX occurred during the multipass hot-rolling process (Fig. 3(f)). The occurrence of DRX is involved in flow softening, which also inhibits DT. The higher frequency of SFs, relative to DTs, is thus likely related to the competitive behavior between DT and DRX. Because the flow stress at high temperatures is highly dependent on the deformation conditions, optimizing the processing parameters (e.g., using a lower deformation temperature and a higher strain rate) may lead to an increase in the number of SFs and nanotwins, while manipulating the state of the lattice defects within the fcc γ-matrix may yield a more suitable balance of the multiple strengthening mechanisms for overcoming the strength–ductility tradeoff.

The SF strengthening mechanism discussed here assumes the cutting of SFs by dislocations, assuming its contribution to be proportional to 1/*L*_*SF*_^16^. In contrast, the GB or TB strengthening mechanisms are explained in terms of dislocation pile-ups, resulting in the Hall-Petch relationship^33, 46^. That is, the interaction with the dislocations differentiates the SF strengthening from the GB and TB strengthening mechanisms. From a practical viewpoint, it is generally difficult to attain homogeneous ultrafine-grained microstructures or nanostructures through conventional manufacturing procedures. Therefore, severe plastic deformation (SPD) techniques, such as equal channel angular pressing (ECAP) and high-pressure torsion (HPT)^55–58^, are generally employed. Similarly, nanotwinned metals are produced by means of electrodeposition^47, 59^, sputtering^60, 61^, or dynamic plastic deformation^62^. Therefore, it is still difficult to fully employ these two mechanisms in bulk forms via industrial production. The SF strengthening, in contrast, can be applied to conventional manufacturing processes and realizes significant strengthening, as demonstrated in the present study. Applicability to industrial production is a major advantage of SF strengthening.

Nanostructured materials often exhibit unusual deformation behaviors, such as large elastic strain^63^ and grain-boundary-mediated plasticity^64, 65^. It is well known that lattice defects in the parent γ-matrix can act as precursors of ε-martensite. Because this strain-induced γ → ε martensitic transformation is responsible for the strain-hardening behavior of biomedical Co–Cr–Mo alloys^66–69^, these lattice defects in the parent γ-phase might enhance the strain-hardening ability of an alloy (see Fig. 1(b)), thus avoiding the early onset of plastic instability and leading to good ductility. Furthermore, SFs themselves act as a barrier against gliding dislocations, in the same was as TBs^20^. The dislocation–SF interactions and the correlation between the preexisting lattice defects and tensile deformation behavior will be described in a follow-up paper.

In conclusion, we conducted multipass hot-rolling of a biomedical Co–Cr–Mo alloy with a single fcc structure and investigated the origin of its significant strengthening based on a quantitative examination of microstructure evolution by electron microscopy and XRD. The strength after multipass hot-rolling linearly increased with *r* and eventually reached approximately 1400 MPa for the 0.2% proof stress at *r* = 90% while maintaining a large elongation of 12%. Grain refinement due to DRX and the development of deformation structures occurred simultaneously during the multipass hot-rolling, although the observed reduction in grain size had much less influence on the overall strength. XRD line-profile analyses revealed that the dislocation density of the initial solution-treated specimen (1.2 × 10^14^ m^−2^) first strongly increased with *r* and then became saturated at a very high dislocation density of 8.5 × 10^15^ m^−2^. The increased dislocation density caused the significant strengthening observed in the hot-rolled specimen. Notably, by using eCMWP analysis, we demonstrated a dramatic increment in the SFP during the multipass hot-rolling process. This corresponds to the significant reduction in *L*_*SF*_ to approximately 10 nm at *r* = 90%. Consequently, the contribution of SFs to overall strengthening became significant at greater values of *r*, although it was not evident at lower *r*. The quantitative evaluation of strengthening mechanisms revealed the first direct evidence of “stacking fault strengthening” in biomedical Co–Cr–Mo alloys and described the manipulation of SFs via thermomechanical processing, which will help in guiding the design and manufacturing strategy for high-strength Co–Cr–Mo alloys.

## Methods

### Sample preparation

A biomedical Co–28Cr–6Mo–0.13 N (Cr: 28.20, Mo: 5.92, N: 0.126, C: 0.04, Mn: 0.62, Si: 0.50, Co: bal. (wt%)) alloy, which satisfies the specifications of the ASTM F1537 standard, was prepared by conventional high-frequency induction melting. An ingot of the alloy weighing around 30 kg (around 150 mm in diameter) was subjected to homogenizing heat treatment and then hot-forged at 1473 K. The alloy was then solution-treated at 1473 K for 15 min, followed by forging at 1473 K to produce a plate specimen with a thickness (*t*) of 15 mm. The specimens for hot-rolling (25 mm in width, 35 mm in length, and 15 mm in thickness) were cut from the plates with an electric discharge machine (EDM) and subjected to solution heat treatment at 1473 K for 10 min. Multipass hot-rolling was conducted with the solution-treated specimens. The specimens were heated to 1473 K and then rolled to produce a height reduction of around 1 mm per pass. This process was repeated to obtain cumulative height reductions (*r*) of 30%, 60%, and 90%. The *r* values were converted to equivalent strains (*ε*_*eq*_) by using the following equation:

8 $${\varepsilon }_{eq}=-\frac{2}{\sqrt{3}}\,{\rm{l}}{\rm{n}}(1 - \frac{r}{100}).$$

To maintain a sufficiently high specimen temperature to prevent fractures during deformation, interval heating was performed at 1473 K for 5 min every two passes (plate thickness *t* ≥ 10 mm) or every pass (*t* ≤ 10 mm). The specimens were air-cooled after the hot-rolling.

### Tensile tests

Uniaxial tensile tests of the initial and hot-rolled specimens were performed at room temperature. Flat samples with a gauge section of 2.0 × 1.0 mm^2^ and gauge length of 10.5 mm were prepared by EDM with the longitudinal axis parallel to the rolling direction (RD). The specimens were strained to failure at a nominal strain rate of 1.6 × 10^−4^ s^−1^. At least three samples for each material were subjected to the tensile test to confirm reproducibility.

### Microstructure observations

Scanning electron microscopy (SEM) observations and electron backscatter diffraction (EBSD) analyses were conducted with a JEOL JSM-7100F and an FEI XL30S-FEG operated at 15 kV and 20 kV, respectively. EBSD data were collected with a step size of 0.2 µm and analyzed using a TSL OIM system. The observations were made on the planes parallel to the rolling plane and at the middle thickness of the rolled specimens. Samples for the above microstructural analyses were ground with emery paper, polished with a 1-µm alumina suspension, and then finished with a 0.04-µm colloidal silica suspension. Transmission electron microscopy (TEM) observations were made using an FEI TITAN^3^ G2 60-300 S/TEM operating at 300 kV with a double spherical aberration (Cs) corrector. The TEM samples were prepared by cutting a 3-mm-diameter disk from each alloy specimen and then grinding it to form a thin film using a dimple grinder (Gatan Model 656). Next, we prepared thin foils through ion-beam milling of the disks (Gatan Model 691, PIPS).

### X-ray diffraction (XRD) line-profile analyses

XRD measurements were performed on the initial and hot-rolled specimens by using an X-ray diffractometer (D8 ADVANCE, Bruker AXS) equipped with a Cu-Kα radiation source. The Bragg–Brentano (2*θ*–*θ*) geometry was employed for obtaining line profiles for the eCMWP analyses. Because both the X-ray scattering of white X-rays and the Cu-Kα_2_ component distort the line profile, incident X-rays were monochromated to the Cu-Kα_1_ line (0.15406 nm) with a Johansson-type monochromator having a quartz-101 reflection. Instrumental line profiles (*I*_*i*_) were obtained from an annealed copper specimen^70^. The eCMWP fitting was performed using CMWP-fit, an open-source software package. The details of the eCMWP method are briefly described in the Supplementary Information.

## Electronic supplementary material

Supplementary Information
