# Supplementary material for: Stacking-fault strengthening of biomedical Co–Cr–Mo alloy via multipass thermomechanical processing
Source: Sci Rep. 2017 Sep 7;7:10808. doi: 10.1038/s41598-017-10305-1 (PMC5589849; doi:10.1038/s41598-017-10305-1)
Supplement: Supplementary file 1 — Supplementary Information [file 41598_2017_10305_MOESM1_ESM.pdf]

Supplementary Information for

Stacking-fault strengthening of biomedical Co−Cr−Mo alloy via multipass thermomechanical processing

Kenta Yamanaka[a,*,†], Manami Mori[b,*], Shigeo Sato[c], Akihiko Chiba[a]

[a]Institute for Materials Research, Tohoku University, 2-1-1 Katahira, Aoba-ku, Sendai 980-8577, Japan

[b]Department of Materials and Environmental Engineering, National Institute of Technology, Sendai College, 48 Nodayama, Medeshima-Shiote, Natori 981-1239, Japan

[c]Graduate School of Science and Engineering, Ibaraki University, 4-12-1 Nakanarusawa, Hitachi 316-8511, Japan

*Equally contributed authors

†Corresponding author: Kenta Yamanaka
Institute for Materials Research, Tohoku University
2-1-1 Katahira, Aoba-ku, Sendai 980-8577, Japan
Tel.: +81 22 215 2118
Fax: +81 22 215 2116
E-mail address: k_yamanaka@imr.tohoku.ac.jp

X-ray diffraction (XRD) line-profile analyses based on the extended convolutional multiple whole profile (eCMWP) fitting

In this study, we used the extended convolutional multiple whole-profile fitting (eCMWP) procedure[1], which was developed based on the CMWP method[2,3], to evaluate the evolution of the dislocation structures and stacking faults during multipass hot rolling. With this method, the measured XRD profiles were fitted by using the convolution profile I:

$$I = I_s \otimes I_m \otimes I_{planar} \otimes I_i \qquad \text{(S1)}$$

where I_s is the function of the size of the smallest unit of a crystal aggregation (i.e., crystallites), I_m is the function of the microstrain that is related to the dislocation density, I_{planar} is the function of planar defects (i.e., stacking faults), and I_i is the function of instrumental effect. The crystallite size is defined as the size of domains that coherently diffract incident X-rays[2] and that are divided by dislocation arrays, subgrains, cell structures, and stacking faults, etc. Therefore, grains that can be observed by microscopy are generally aggregates of these small domains, and the grain sizes are different from the crystallite sizes. The area-averaged crystallite size D can be calculated as

$$D = m\ \exp(2.5\sigma^2), \qquad \text{(S2)}$$

where m is the median and σ is the size distribution function. I_m is given by its Fourier transform, which is derived as[4,5]

$$I_m = \exp\left(-2\pi^2 g^2 L^2 \langle \varepsilon^2_{\mathrm{g,L}} \rangle\right), \qquad \text{(S3)}$$

where g is the absolute value of the diffraction vector and L is the Fourier variable. $\langle \varepsilon^2_{\mathrm{g,L}} \rangle$ is the mean square strain and is defined as[5]

$$\langle \varepsilon^2_{\mathrm{g,L}} \rangle = \left(\rho_{dis}\overline{C}b^2/4\pi\right) f(L/R_e), \qquad \text{(S4)}$$

where ρ_{dis}, $\overline{C}$, b, f, and R_e are the dislocation density, contrast factor of dislocations, absolute value of the Burgers vector, Wilkens function, and effective outer cutoff radius of dislocations, respectively[4–6]. The function $f(L/R_e)$ was determined for distributions of dislocations in the entire L range from zero to infinity[5]. The dislocation arrangement can be evaluated using the eCMWP fitting with a suitable dimensionless parameter M[7]:

$$M = R_e\sqrt{\rho_{dis}}. \qquad \text{(S5)}$$

Given that dislocations are a major source of microstrain in a cubic polycrystalline system, the contrast factors of dislocations $\overline{C}$ can be averaged over the permutations of the hkl indices and defined as[4]

$$\overline{C} = \overline{C}_{h00}\left(1 - qH^2\right),\qquad \text{(S6)}$$

where q is a parameter depending on the slip system and elastic properties of the crystal, $\overline{C}_{h00}$ is the average contrast factor corresponding to the $h00$ reflection, and a is the lattice constant.

The eCMWP procedure was used to numerically model I_{planar} in Equation S1 in accordance with the theory proposed by Warren[8] and developed by Treacy *et al.*[9]. According to this theory, planar defects shift and broaden the diffraction profiles corresponding to $h + k + l \neq 3n$, where n is the integer value, but have no effect on the other profiles. In the eCMWP procedure, each profile is divided into five sub-reflections at most. One of them is written as a delta function, and the others are modeled in terms of Lorentzian functions:

$$I_{planar} = w_\delta I_\delta + \sum_{j=0}^{4} w_L^j I_L^j,\qquad \text{(S7)}$$

where I_δ and I_L are the delta function at the Bragg position and the Lorentzian profile function, respectively. In this approach, the full width at half maximum (FWHM) and position of the Lorentzian profile function are expanded by the fifth-order polynomial:

$$FWHM_L^j = \sum_{n=1}^{5} a_n P_{SF}^n\qquad \text{(S8)}$$

$$shift_L^j = \sum_{n=1}^{5} b_n P_{SF}^n,\qquad \text{(S9)}$$

where P_{SF} is the fault probability and a_n and b_n are fifth-order polynomial coefficients of each type of planar defects.

The procedures for the eCMWP fitting were carried out using CMWP-fit, an open source software, available from the webpage: http://csendes.elte.hu/cmwp/.

References

1. Balogh, L., Ribárik, G. & Ungár, T. Stacking faults and twin boundaries in fcc crystals determined by x-ray diffraction profile analysis. *J. Appl. Phys.* **100,** 23512 (2006).
2. Ungár, T., Gubicza, J., Ribárik, G. & Borbély, A. Crystallite size distribution and dislocation structure determined by diffraction profile analysis: Principles and practical application to cubic and hexagonal crystals. *J. Appl. Crystallogr.* **34,** 298–310 (2001).
3. Ribárik, G., Gubicza, J. & Ungár, T. Correlation between strength and microstructure of ball-milled Al–Mg alloys determined by X-ray diffraction. *Mater. Sci. Eng. A* **387−389,** 343–347 (2004).

4. Ungár, T. & Tichy, G. The Effect of Dislocation Contrast on X-Ray Line Profiles in Untextured Polycrystals. *Phys. Status Solidi* **171,** 425–434 (1999).
5. Wilkens, M. Theoretical aspects of kinematical X-ray diffraction profiles from crystals containing dislocation distributions. in *Fundamental Aspects of Dislocation Theory: Conference Proceedings, National Bureau of Standards, April 21−25, 1969* (eds. Simmons, J., DeWit, R. & Bullough, R.) 1195–1221 (U.S. National Bureau of Standards, 1970).
6. Ribárik, G., Ungár, T. & Gubicza, J. MWP-fit: A program for multiple whole-profile fitting of diffraction peak profiles by ab initio theoretical functions. *J. Appl. Crystallogr.* **34,** 669–676 (2001).
7. Wilkens, M. The mean square stress <σ2> for restrictedly random distributions of dislocations in a cylindrical body. *Acta Metallurgica* **17,** 1155–1159 (1969).
8. Warren, B. E. *X-ray Diffraction*. (Addison-Wesley, 1969).
9. Treacy, M. M. J., Newsam, J. M. & Deem, M. W. A General Recursion Method for Calculating Diffracted Intensities from Crystals Containing Planar Faults. *Proceedings of the Royal Society A: Mathematical, Physical and Engineering Sciences* **433,** 499–520 (1991).
